# Effects of Quinine, Quinidine and Chloroquine on Human Muscle Nicotinic Acetylcholine Receptors

**DOI:** 10.3389/fphar.2018.01339

**Published:** 2018-11-20

**Authors:** Günter Gisselmann, Desiree Alisch, Brigitte Welbers-Joop, Hanns Hatt

**Affiliations:** ^1^Department of Cell Physiology, Ruhr-University-Bochum, Bochum, Germany; ^2^Cassella-med GmbH & Co. KG, Koeln, Germany

**Keywords:** quinine, nocturnal leg cramps, nicotinic acetylcholine receptor, two-electrode voltage clamp, ion channel block

## Abstract

The genus *Cinchona* is known for a range of alkaloids, such as quinine, quinidine, cinchonine, and cinchonidine. *Cinchona* bark has been used as an antimalarial agent for more than 400 years. Quinine was first isolated in 1820 and is still acknowledged in the therapy of chloroquine-resistant falciparum malaria; in lower dosage quinine has been used as treatment for leg cramps since the 1940s. Here we report the effects of the quinoline derivatives quinine, quinidine, and chloroquine on human adult and fetal muscle nicotinic acetylcholine receptors (nAChRs). It could be demonstrated that the compounds blocked acetylcholine (ACh)-evoked responses in *Xenopus laevis* oocytes expressing the adult nAChR composed of αβ𝜀δ subunits in a concentration-dependent manner, with a ranked potency of quinine (IC_50_ = 1.70 μM), chloroquine (IC_50_ = 2.22 μM) and quinidine (IC_50_ = 3.96 μM). At the fetal nAChR composed of αβγδ subunits, the IC_50_ for quinine was found to be 2.30 μM. The efficacy of the block by quinine was independent of the ACh concentration. Therefore, quinine is proposed to inhibit ACh-evoked currents in a non-competitive manner. The present results add to the pharmacological characterization of muscle nAChRs and indicate that quinine is effective at the muscular nAChRs close to therapeutic blood concentrations required for the therapy and prophylaxis of nocturnal leg cramps, suggesting that the clinically proven efficacy of quinine could be based on targeting nAChRs.

## Introduction

**FIGURE 1:**
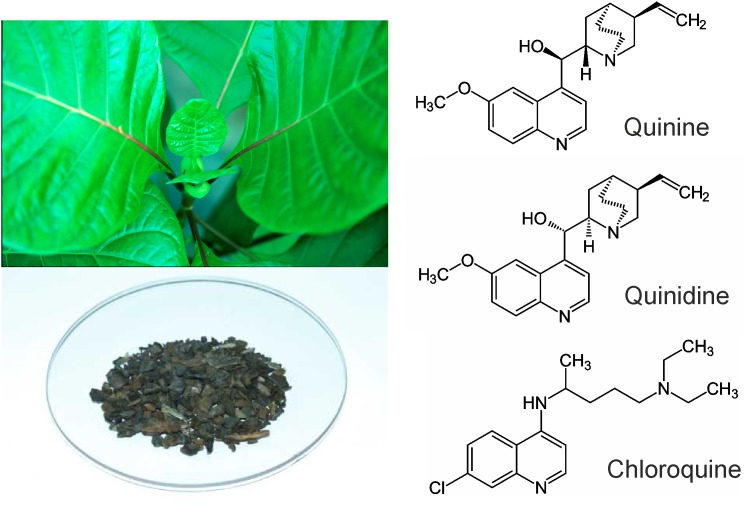
Left: *Cinchona calisaya* and *Cinchona* bark as the source for quinine. Right: Molecular structures of quinine, quinidine, and chloroquine.

Quinoline derivatives such as natural quinine, quinidine, and synthetically produced chloroquine are well known for their use in the treatment of malaria (Figure 1). The efficacy of quinine against nocturnal leg cramps has been proven in randomized clinical trials (Diener et al., 2002; El-Tawil et al., 2015). But the muscle relaxant mechanism of action of quinine has not been fully elucidated yet. As it does not freely cross the blood brain barrier (Silamut et al., 1985), quinine is supposed to be a peripheral muscle relaxant *in vivo*. In higher dosage as used against malaria, central nervous effects may occur. Quinine and its derivatives are acting on a variety of ion channels including several types of potassium channels (Glavinović and Trifaró, 1988; Imai et al., 1999), members of family of ligand-gated ion channels such as the 5-HT3-type of serotonin receptor (Thompson et al., 2007; Thompson and Lummis, 2008) and nicotinic acetylcholine receptors (nAChR) (Fukudome et al., 1998b; Ballestero et al., 2005). Quinine’s significant bitterness, widely enjoyed in tonic bitter lemonades, is attributed to activation of bitter taste receptors (T2Rs) which are not limited to taste buds but are expressed in many extraoral tissues (Upadhyaya et al., 2016). Further, quinolines influence cholinergic synaptic transmission (Sieb et al., 1996). This effect is possibly the basis for their use in further applications like the treatment of muscle cramps (Katzberg, 2015) and slow channel congenital myasthenic syndrome (SCCMS) (Harper and Engel, 1998; Harper et al., 2003; Peyer et al., 2013).

It is known that quinine acts on muscular and neuronal nAChRs (Sieb et al., 1996; Fukudome et al., 1998a,b; Ballestero et al., 2005). However, the interaction of quinine has also been reported for receptors present at the neuromuscular junction, in which it produces long-lived open-channel as well as a closed-channel block and can normalize the open duration of channel events in the slow-channel congenital myasthenic syndrome (Sieb et al., 1996; Fukudome et al., 1998a,b). Slow channel congenital myasthenic syndrome (SCCMS) is caused by missense mutations in subunits of nicotinic acetylcholine receptor (AChR) at the neuromuscular junction (Engel, 2018). Mutated AChR channel produces prolonged opening events leading to a depolarization block and endplate myopathy. Quinoline derivatives such as quinidine correct the prolonged opening times of the mutated acetylcholine receptor channels in myasthenic syndrome (Bleecker et al., 1998). On the contrary, in myasthenia gravis quinine is contraindicated because it decreases the excitability of the motor end-plate region, thereby reducing responses to repetitive nerve stimulation by acetylcholine. These may be the underlying effects explaining the use of low dose quinine in the therapy and prophylaxis of leg cramps.

Quinine is also blocker for the neuronal α9α10 nAChRs. At this cochlear type of ACh receptor, a mixed competitive and non-competitive inhibition was observed (Ballestero et al., 2005). nAChRs form together with 5-HT3-, GABA(A)-, and glycine receptors the superfamily of ligand-gated ion channels (Albuquerque et al., 2009). Possibly due the strong structural and functional similarities between these receptors, quinine is also a blocker of 5-HT3- and at elevated concentrations also of GABA(A) receptors (Thompson and Lummis, 2008).

The composition of muscle nAChRs is dependent of the developmental stage. The fetal nAChR together with the receptor in denervated muscles is composed of αβγδ subunits with a stoichiometry of (α1)_2_β_1_γδ. In the adult, γ is replaced by the 𝜀 subunit (Kalamida et al., 2007). In our present study, we examined the effects of the quinoline derivatives quinine, quinidine, and chloroquine on adult and fetal human muscle nAChRs recombinantly expressed in *X. laevis* oocytes and provide evidence that these compounds block the nAChRs.

## Materials and Methods

### Expression System

The expression plasmids contain the cDNA coding for the different human nAChR subunits in pRBG4 (Ohno et al., 1996). Cloned muscular AChR subunit cDNAs were kindly provided by Dr A. G. Engel (Mayo Clinic, Rochester, MN, United States) and Dr. F. Grassi (Carattere Scientifico San Raffaele Pisana, Rome, Italy). Cloned cDNA of the human a7 AChR subunit was described by Schreiner et al. (2014). cRNAs were prepared using the AmpliCap T3 high-yield message marker kit (Epicenter, Madison, WI, United States), following the manufacturer’s protocol. Oocytes were obtained as previously described (Gisselmann et al., 2004) and injected with a total amount of ∼30 ng of a mixture of the receptor-coding cRNA using an injection-setup from WPI (Nanoliter 2000, Micro4). For the muscular type, a mixture of cRNAs for α, β, δ, and 𝜀 in a stoichiometry of 2:1:1:1 was used and for the fetal type, 𝜀 was replaced by γ. The injected oocytes were stored in ND 96 (96.0 mM NaCl, 2.0 mM KCl, 1.8 mM CaCl_2_, 1.0 mM MgCl_2_, 5.0 mM HEPES, pH 7.2, 200 U/ml penicillin, and 200 μg/ml streptomycin) at 12 °C. Measurements were performed 4–6 days after cRNA injection.

### Electrophysiology

The electrophysiological recordings were performed using the two-electrode voltage clamp technique as previously described (Sergeeva et al., 2010). All of the measurements were performed in normal frog ringer (NFR) [115 mM NaCl, 2.5 mM KCl, 1.8 mM CaCl_2_, 10 mM HEPES; pH 7.2 (NaOH/HCl)]. The currents were recorded at a holding potential of typically -60 mV using the Cell Works 6.1.1. software (NPI).

### Substances

The chemicals were obtained from Sigma-Aldrich or Cassella-med GmbH & Co. KG (quinine) and disssolved in NFR.

### Data Analysis

In blocking experiments, the test substances were applied in an alternating manner with ACh. Therefore, the currents of the test substances or the modulated currents were normalized to the mean of the ACh-induced currents before and after the test substance was applied. The concentration-response data were fitted with the logistic equation using SigmaPlot 8.0 (SPSS). The deviations are represented by the standard error of the mean (SEM).

## Results

### Effect of Quinoline Derivatives on Adult Muscle nAChRs

**FIGURE 2:**
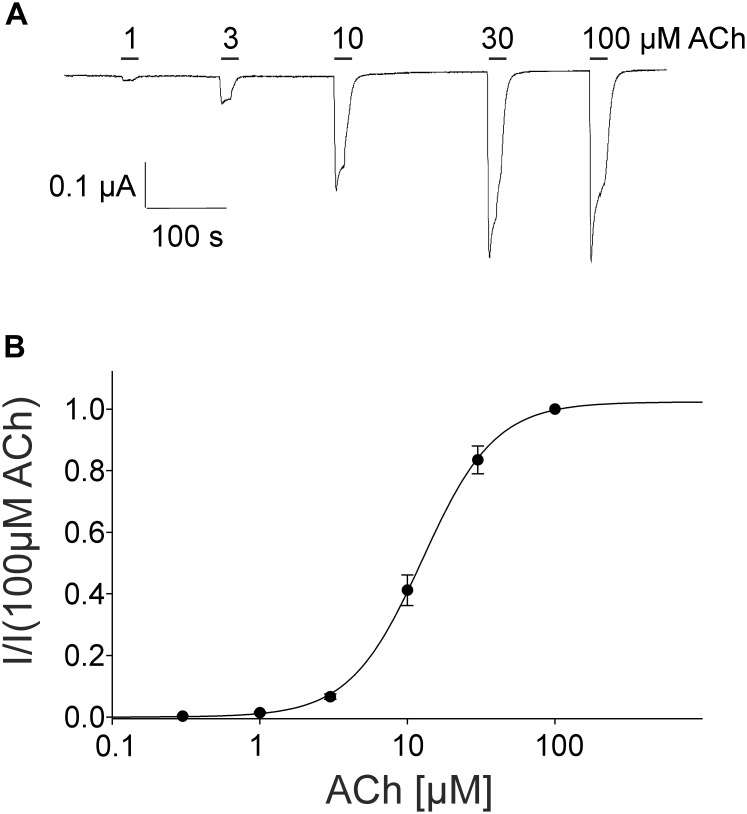
Concentration-dependent activating effect of ACh on the adult human nicotinic acetylcholine receptor αβ𝜀δ measured in *Xenopus* oocytes. **(A)** Representative membrane currents evoked by ACh measured by two-electrode voltage-clamp. The mean current evoked by 10 μM at the third application was 95% +-. **(B)** Concentration–response curve of the receptor activated by different concentrations of acetylcholine (*n* = 6). Holding potential: –60 mV, error bars represent S.E.M.

**FIGURE 3:**
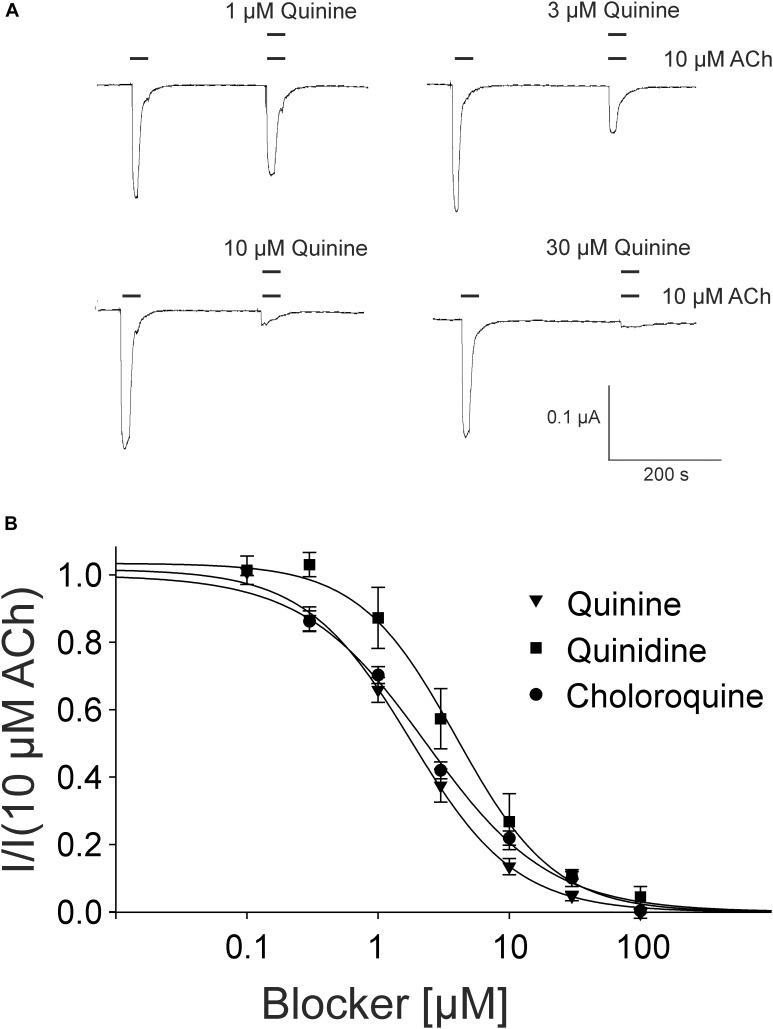
Block of adult muscle nicotinic acetylcholine receptor by quinidine and derivatives in *Xenopus* oocytes. **(A)** Representative membrane currents measured by two-electrode voltage-clamp. Currents were elicited by 10 μM ACh in presence of quinine. **(B)** Concentration–inhibition curves for quinine (inverted triangles), quinidine (squares), or chloroquine (circles) at ACh mediated currents elicited by 10 μM ACh in the presence of different concentrations of the blockers (*n* = 6). Holding potential: –60 mV, error bars represent S.E.M.

For the pharmacological characterization, we expressed the human muscle nAChRs recombinantly in *X. laevis* oocytes and characterized the ion channel activity using the two-electrode voltage-clamp technique. In the first experiments, we established a concentration response curve for ACh on *Xenopus* oocytes expressing the adult nAChR composed of αβγ𝜀 subunits (Figure 2). Under our experimental conditions, an EC_50_ value for ACh of 12.65 ± 2.40 μM, n_H_ = 1.77 ± 0.05 (*n* = 6) was determined, similar to the EC_50_ values previously reported in this expression system (Jonsson Fagerlund et al., 2009). Next, we tested the modulatory effects of quinoline derivatives on the response evoked by 10 μM ACh, a near EC_50_ value of the agonist. All three tested quinoline derivatives blocked the ACh-evoked currents in a dose-dependent manner with a potency in the low μM range: quinine was most potent with an IC_50_ of 1.70 ± 0.12 μM (*n* = 6) followed by chloroquine with an IC_50_ of 2.22 ± 0.19 μM (*n* = 6). Quinidine was somehow less potent and blocked with an IC_50_ of 3.96 ± 0.36 μM (*n* = 6) (Figure 3). The block by quinine was reversible, as shown in the Supplementary Figure 1A.

### Non-competitive Action of Quinine

**FIGURE 4:**
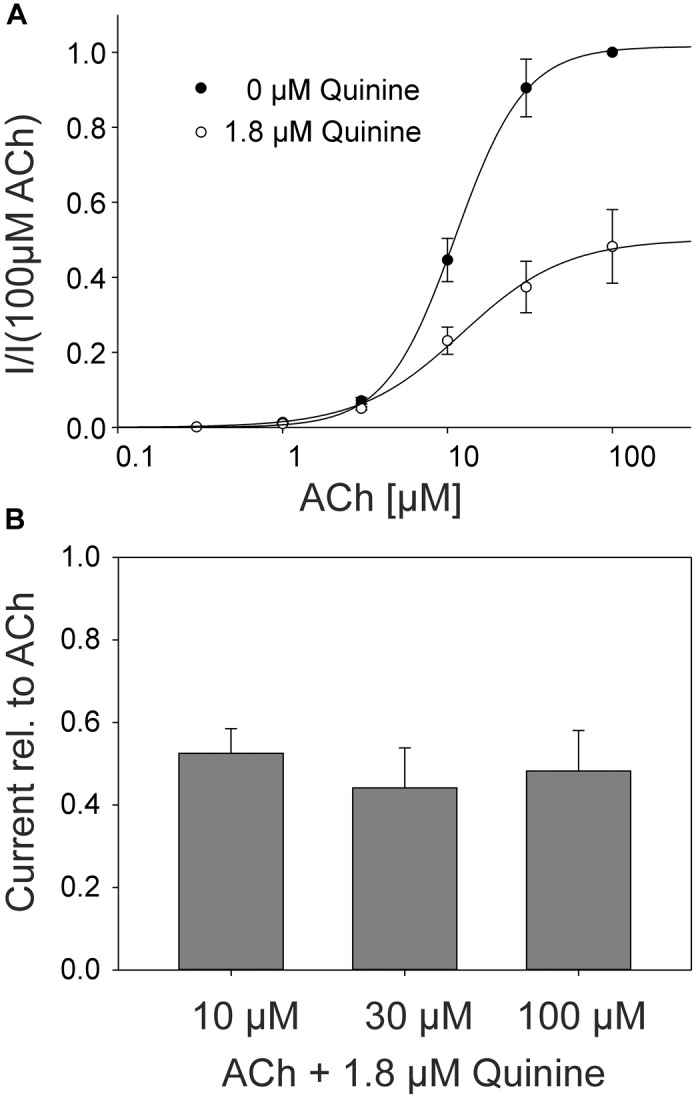
ACh concentration–response relationship at human adult muscle nAChRs in the presence and absence of 1.8 μM quinine measured in *Xenopus* oocytes. Currents were elicited by various ACh concentrations. **(A)** Concentration-response curves for human adult muscle nAChRs in the presence (open circles) and the absence (filled circles) of 1.8 μM quinine (*n* = 6). **(B)** Relative current blocked by 1.8 μM quinine at 10, 30, and 100 μM ACh. Holding potential: -60 mV, error bars represent S.E.M.

In the rat α9α10 nAChRs, a mixed competitive / non-competitive mode of antagonism for quinine was observed (Ballestero et al., 2005). To determine the mode of antagonism for quinine in the adult muscle nAChRs, we established concentration-response curves for ACh alone and for different concentrations of ACh in the presence of quinine at the same *Xenopus* oocyte. We used 1.8 μM quinine which is a near IC_50_ concentration. In the case of a non-competitive mechanism, the inhibition should be independent of the ACh concentration, whereas the efficacy of competitive antagonists decreases with increasing ACh concentrations. The efficacy of quinine was independent of the ACh concentration (Figure 4A) and the determined EC_50_ of ACh didn’t significantly change in the presence of 1.8 μM quinine (Figure 4B). Further, also at elevated ACh concentrations, the maximally evoked current was never reached in the presence of 1.8 μM quinine (Figure 4B).

For the rat α9α10 nAChRs a slight voltage dependency of the quinine block was described indicating a potential binding site within or near the channel pore (Ballestero et al., 2005). We tested whether the quinine block of the muscular AChR was also voltage dependent and found that 1.8 μM quinine blocked more pronounced at negative (-60 mV, I/I_max_ = 0.45 ± 0.11, *n* = 3) than at positive membrane potentials (++20 mV, I/I_max_ = 0.87 ± 0.07, *n* = 3) (Supplementary Figure 1C).

### Effect of Quinine on Fetal Muscle and Neuronal nAChRs

**FIGURE 5:**
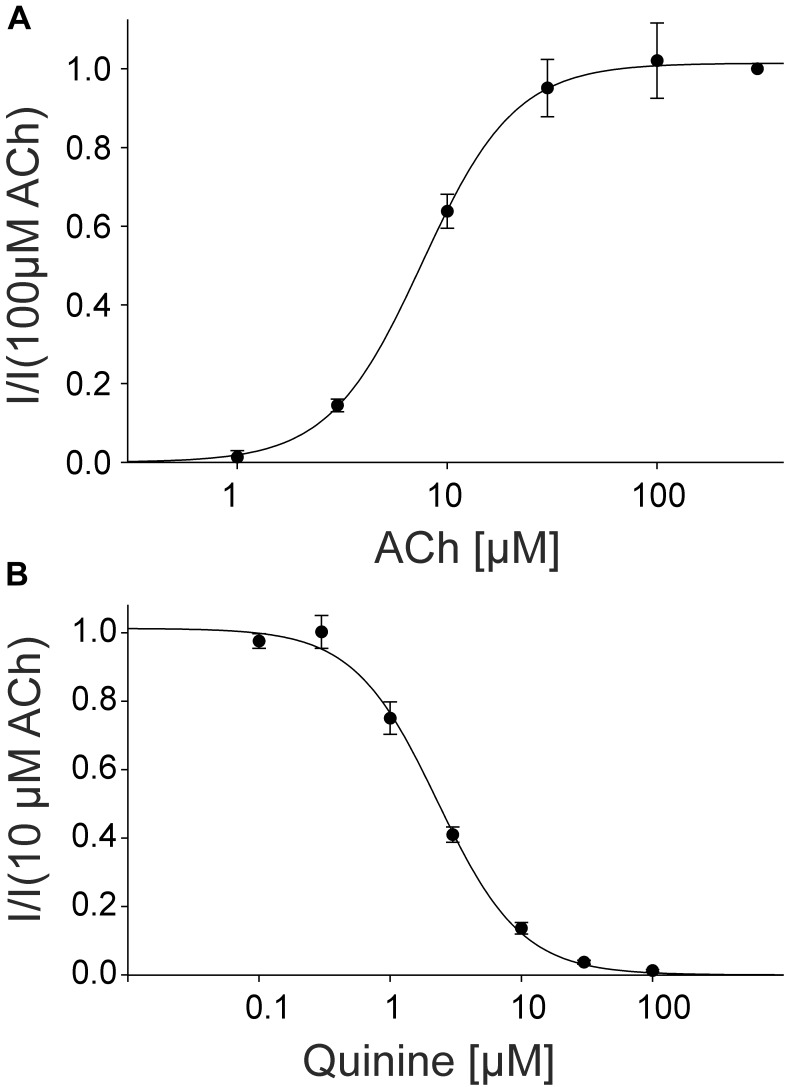
Concentration-dependent activating effect of ACh on the fetal human nicotinic acetylcholine receptor αβγδ measured in *Xenopus* oocytes. **(A)** Concentration–response curve of the receptor activated by different concentrations of acetylcholine (*n* = 6). **(B)** Block of human fetal muscle nAChRs by quinidine in a concentration-dependent manner. Concentration–inhibition curves for quinine (circles) at ACh mediated currents elicited by 10 μM ACh in the presence of different concentrations of the blocker (*n* = 3). Holding potential: -60 mV, error bars represent S.E.M.

For the pharmacological characterization, we expressed the fetal muscle nAChRs composed of αβγδ subunits recombinantly in *X. laevis* oocytes and characterized the ion channels using the two-electrode voltage-clamp technique. In the first experiments, we established a concentration response curve for ACh on *Xenopus* oocytes expressing fetal nAChRs (Figure 5). Under our experimental conditions, an EC_50_ value for ACh of 7.60 ± 1.87 μM, (*n* = 6) was determined, comparable for the EC_50_ reported for fetal receptors in the *Xenopus* oocyte system (Garland et al., 1998). Next, we tested the modulatory effects of quinine on the response evoked by 10 μM ACh, a near EC_50_ value of the agonist, which blocked the ACh-evoked currents in a dose-dependent manner with an IC_50_ of 2.30 ± 0.17 μM (*n* = 3) (Figure 4).

For the pharmacological characterization of neuronal receptor types, we expressed nAChRs composed of α7 subunits in *X. laevis* oocytes. Under our experimental conditions, the EC_50_ value for ACh was previously reported to be 90.5 μM (Schreiner et al., 2014). We tested the modulatory effects of quinine on the response evoked by 100 μM ACh, a near EC_50_ value of the agonist, which blocked the ACh-evoked currents in a dose-dependent manner with an IC_50_ of 12.8 ± 1.3 μM (*n* = 3) (Supplementary Figure 1B). The neuronal α7 subtype is of significantly lower potency compared to the muscular type (*p* < 0.0001).

## Discussion

This study describes the effects of quinine, quinidine, and chloroquine on human muscle nAChRs. All three compounds are potent antagonists which block in the low μ-molar range (IC_50_ ∼ 1.7–4 μM) which corresponds to a concentration of 0.6–1.4 mg/l quinine sulfate. Compared with the reported potency at other receptors, quinine shows a similar potency at the neuronal rat α9α10 nAChRs (IC_50_∼1 μM) and the human homomeric 5HT3_A_-receptors (IC_50_∼1 μM), but is less potent at the human neuronal α7 nAChR (IC_50_∼13 μM), the human heteromeric 5HT3_AB_- (IC_50_∼16 μM) or human GABA(A)-receptors (IC_50_∼1.6 mM) (Ballestero et al., 2005; Thompson and Lummis, 2008). At a similar low μM concentration range the interference with the neuromuscular transmission (Sieb et al., 1996) or the decrease in the open duration of muscle nAChRs (Fukudome et al., 1998b) was observed for quinoline derivatives *in vitro*.

At human muscle nAChRs receptors the observed quinine block was consistent with a non-competitive antagonism. These mode of antagonism for quinine was also reported at human heteromeric 5-HT3_AB_-receptors (Thompson and Lummis, 2008). At rat neuronal α9α10 nAChRs, quinine was proposed to block competitively at lower, however, non-competitively at higher concentrations (Ballestero et al., 2005). We observed a voltage-dependency for the quinine block of the muscular AChR similar to that reported for the rat α9α10 nAChR. Our results indicate that quinine could interact with a charged side chain within or near the channel pore as discussed in detail for the α9α10 nAChR by Ballestero et al. (2005).

Human homomeric 5-HT3_A_ and GABA(A) receptors were blocked in a competitive manner (Thompson and Lummis, 2008). Binding studies at the recombinant muscle receptor expressed in HEK293 cells support the observed non-competitive antagonism of quinine, as even concentrations up to 30 μM of the antagonist could not inhibit the binding of α-bungarotoxin (Sieb et al., 1996).

Regarding the high homology between 5-HT3_A_, GABA(A) and nAChRs and the similarities in the actions of quinoline compounds, conserved sites of action for these compounds were suggested (Thompson and Lummis, 2008).

For the therapy of malaria, an about ten-fold higher oral dosage of quinine is needed than against muscle cramps, and anti-malaria plasma concentrations of 10–15 mg/l are recommended. During acute malaria, quinine plasma concentrations are higher than under non-infectious conditions. Metabolic clearance of quinine in acute illness is reduced because of decreased cytochrome P450 (CYP) 3A4 activity. Increased α-1-acid glycoprotein concentrations in the acute phases of malaria lead to an increased plasma protein binding of quinine, which contributes to the decreased volume of distribution and the increased quinine concentration during the acute phase of the disease (Kloprogge et al., 2014). Thus, the *in vitro* observed effects of quinine at central nervous receptors are to be expected in general above a plasma concentration of 10 μg/ml which is not reached after intake of 200–400 mg quinine sulfate against muscle cramps.

When quinine sulfate 2 × 260 mg is applied orally to humans, a dosage which lies in the range used in therapy and prophylaxis of nocturnal leg cramps, a peak plasma concentration of 2.5 μg/ml is reached at about 2 h after intake of quinine. Plasma elimination half time is about 11–12 h; accordingly, plasma concentration was decreased to 1.6 μg/ml after 12 h (Dieterich et al., 1984). These values are in the same concentration range as measured for the inhibitory effect of quinine at the human muscle AChR; thus, this effect may be postulated as a possible mechanism of action of the muscle relaxant effect of quinine which has been therapeutically used in patients with leg cramps for more than half a century.

In summary, we have shown that the quinine, quinidine and chloroquine antagonize ACh-evoked responses at muscular nAChRs. The potency for quinine at the muscle nAChRs was in the same range as found for neuronal rat α9α10 AChR or human 5-HT3_A_-receptors (Ballestero et al., 2005; Thompson and Lummis, 2008). However, quinine was slightly less potent at the neuronal human α7 AChR.

Typical blood and tissue concentrations for quinine indicate possible action on muscle nAChRs. These observations further extend the pharmacological knowledge on receptors affected by quinoline derivatives. The reversible inhibitory effect of quinine on human muscle nAChRs may be one mechanism which contributes to the clinically proven efficacy of quinine against leg cramps. But as quinine is a wide spectrum channel blocker, further research on its interaction with receptors of different type and location would be of interest.

## Author Contributions

HH, GG, and BW-J conceived and designed the experiments. DA performed the experiments. DA and GG analyzed the data. GG and BW-J wrote the paper.

## Conflict of Interest Statement

BW-J was employed by company Cassella-med GmbH &Co. The remaining authors declare that the research was conducted in the absence of any commercial or financial relationships that could be construed as a potential conflict of interest.

## References

[B1] Albuquerque E. X. Pereira E. F. Alkondon M. Rogers S. W. (2009). Mammalian nicotinic acetylcholine receptors: from structure to function. *Physiol. Rev.* 89 73–120. 10.1152/physrev.00015.2008 19126755 PMC2713585

[B2] Ballestero J. A. Plazas P. V. Kracun S. Gómez-Casati M. E. Taranda J. Rothlin C. V. (2005). Effects of quinine, quinidine, and chloroquine on alpha9alpha10 nicotinic cholinergic receptors. *Mol. Pharmacol.* 68 822–829. 10.1124/mol.105.014431 15955868

[B3] Bleecker J. L. de Meire V. I. Pappens S. (1998). Quinidine prevents paraoxon-induced necrotizing myopathy in rats. *Neurotoxicology*, 19 833–838. 9863772

[B4] Diener H. C. Dethlefsen U. Dethlefsen-Gruber S. Verbeek P. (2002). Effectiveness of quinine in treating muscle cramps: a double-blind, placebo-controlled, parallel-group, multicentre trial. *Int. J. Clin. Pract.* 56 243–246. 12074203

[B5] Dieterich H. A. Messer W. Jaehnchen E. (1984). Plasma concentrations of theophylline and quinine in healthy volunteers following oral administration of a fixed drug combination. *Arzneimittelforschung* 34 520–522. 6540112

[B6] El-Tawil S. Al Musa T. Valli H. Lunn M. P. T. Brassington R. El-Tawil T. (2015). Quinine for muscle cramps. *Cochrane Database Syst. Rev.* 8:CD005044. 10.1002/14651858.CD005044.pub3 25842375 PMC11055607

[B7] Engel A. G. (2018). Genetic basis and phenotypic features of congenital myasthenic syndromes. *Handb. Clin. Neurol.* 148 565–589. 10.1016/B978-0-444-64076-5.00037-5 29478601

[B8] Fukudome T. Ohno K. Brengman J. M. Engel A. G. (1998a). AChR channel blockade by quinidine sulfate reduces channel open duration in the slow-channel congenital myasthenic syndrome. *Ann. N. Y. Acad. Sci.* 841 199–202. 10.1111/j.1749-6632.1998.tb10928.x 9668240

[B9] Fukudome T. Ohno K. Brengman J. M. Engel A. G. (1998b). Quinidine normalizes the open duration of slow-channel mutants of the acetylcholine receptor. *Neuroreport* 9 1907–1911. 9665624 10.1097/00001756-199806010-00044

[B10] Garland C. M. Foreman R. C. Chad J. E. Holden-Dye L. Walker R. J. (1998). The actions of muscle relaxants at nicotinic acetylcholine receptor isoforms. *Eur. J. Pharmacol* 357 83–92. 10.1016/S0014-2999(98)00542-1 9788777

[B11] Gisselmann G. Plonka J. Pusch H. Hatt H. (2004). Unusual functional properties of homo- and heteromultimeric histamine-gated chloride channels of *Drosophila melanogaster*: spontaneous currents and dual gating by GABA and histamine. *Neurosci. Lett.* 372 151–156. 10.1016/j.neulet.2004.09.031 15531107

[B12] Glavinović M. I. Trifaró J. M. (1988). Quinine blockade of currents through Ca2+-activated K+ channels in bovine chromaffin cells. *J. Physiol.* 399 139–152. 10.1113/jphysiol.1988.sp017072 2457086 PMC1191656

[B13] Harper C. M. Engel A. G. (1998). Quinidine sulfate therapy for the slow-channel congenital myasthenic syndrome. *Ann. Neurol.* 43 480–484. 10.1002/ana.410430411 9546329

[B14] Harper C. M. Fukodome T. Engel A. G. (2003). Treatment of slow-channel congenital myasthenic syndrome with fluoxetine. *Neurology* 60 1710–1713. 10.1212/01.WNL.0000061483.11417.1B 12771277

[B15] Imai S. Suzuki T. Sato K. Tokimasa T. (1999). Effects of quinine on three different types of potassium currents in bullfrog sympathetic neurons. *Neurosci. Lett.* 275 121–124. 10.1016/S0304-3940(99)00775-2 10568514

[B16] Jonsson Fagerlund M. Dabrowski M. Eriksson L. I. (2009). Pharmacological characteristics of the inhibition of nondepolarizing neuromuscular blocking agents at human adult muscle nicotinic acetylcholine receptor. *Anesthesiology* 110 1244–1252. 10.1097/ALN.0b013e31819fade3 19417616

[B17] Kalamida D. Poulas K. Avramopoulou V. Fostieri E. Lagoumintzis G. Lazaridis K. (2007). Muscle and neuronal nicotinic acetylcholine receptors. Structure, function and pathogenicity. *FEBS J.* 274 3799–3845. 10.1111/j.1742-4658.2007.05935.x 17651090

[B18] Katzberg H. D. (2015). Neurogenic muscle cramps. *J. Neurol.* 262 1814–1821. 10.1007/s00415-015-7659-x 25673127

[B19] Kloprogge F. Jullien V. Piola P. Dhorda M. Muwanga S. Nosten F. (2014). Population pharmacokinetics of quinine in pregnant women with uncomplicated *Plasmodium falciparum* malaria in uganda. *J. Antimicrob. Chemother.* 69 3033–3040. 10.1093/jac/dku228 24970740 PMC4195470

[B20] Ohno K. Wang H. L. Milone M. Bren N. Brengman J. M. Nakano S. (1996). Congenital myasthenic syndrome caused by decreased agonist binding affinity due to a mutation in the acetylcholine receptor epsilon subunit. *Neuron* 17 157–170. 10.1016/S0896-6273(00)80289-5 8755487

[B21] Peyer A.-K. Abicht A. Heinimann K. Sinnreich M. Fischer D. (2013). Quinine sulfate as a therapeutic option in a patient with slow channel congenital myasthenic syndrome. *Neuromuscul. Disord.* 23 571–574. 10.1016/j.nmd.2013.04.001 23688972

[B22] Schreiner B. S. Lehmann R. Thiel U. Ziemba P. M. Beltrán L. R. Sherkheli M. A. (2014). Direct action and modulating effect of (+)- and (-)-nicotine on ion channels expressed in trigeminal sensory neurons. *Eur. J. Pharmacol.* 728 48–58. 10.1016/j.ejphar.2014.01.060 24512725

[B23] Sergeeva O. A. Kletke O. Kragler A. Poppek A. Fleischer W. Schubring S. R. (2010). Fragrant dioxane derivatives identify beta1-subunit-containing GABAA receptors. *J. Biol. Chem.* 285 23985–23993. 10.1074/jbc.M110.103309 20511229 PMC2911342

[B24] Sieb J. P. Milone M. Engel A. G. (1996). Effects of the quinoline derivatives quinine, quinidine, and chloroquine on neuromuscular transmission. *Brain Res.* 712 179–189. 10.1016/0006-8993(95)01349-0 8814892

[B25] Silamut K. White N. J. Looareesuwan S. Warrell D. A. (1985). Binding of quinine to plasma proteins in falciparum malaria. *Am. J. Trop. Med. Hyg.* 34 681–686. 10.4269/ajtmh.1985.34.681 3896000

[B26] Thompson A. J. Lochner M. Lummis S. C. (2007). The antimalarial drugs quinine, chloroquine and mefloquine are antagonists at 5-HT3 receptors. *Br. J. Pharmacol.* 151 666–677. 10.1038/sj.bjp.0707238 17502851 PMC1994240

[B27] Thompson A. J. Lummis S. C. R. (2008). Antimalarial drugs inhibit human 5-HT(3) and GABA(A) but not GABA(C) receptors. *Br. J. Pharmacol.* 153 1686–1696. 10.1038/bjp.2008.34 18311193 PMC2438262

[B28] Upadhyaya J. D. Chakraborty R. Shaik F. A. Jaggupilli A. Bhullar R. P. Chelikani P. (2016). The pharmacochaperone activity of quinine on bitter taste receptors. *PLoS One* 11:e0156347. 10.1371/journal.pone.0156347 27223611 PMC4880206

