# Supplementary material for: Effects of Quinine, Quinidine and Chloroquine on Human Muscle Nicotinic Acetylcholine Receptors
Source: Front Pharmacol. 2018 Nov 20;9:1339. doi: 10.3389/fphar.2018.01339 (PMC6255974; doi:10.3389/fphar.2018.01339)
Supplement: Supplementary file 1 [file Data_Sheet_1.PDF]

SUPPLEMENTARY MATERIAL

Effects of quinine, quinidine and chloroquine on human muscle nicotinic acetylcholine receptors

Günter Gisselmann[1], Desiree Alisch[1], Brigitte Welbers-Joop[2], Hanns Hatt[1*]

1 Department of Cell Physiology, Ruhr-University-Bochum, Bochum, Germany.

2 Cassella-med GmbH & Co. KG, Koeln, Germany

*Correspondence: Hanns Hatt, hans.hatt@rub.de

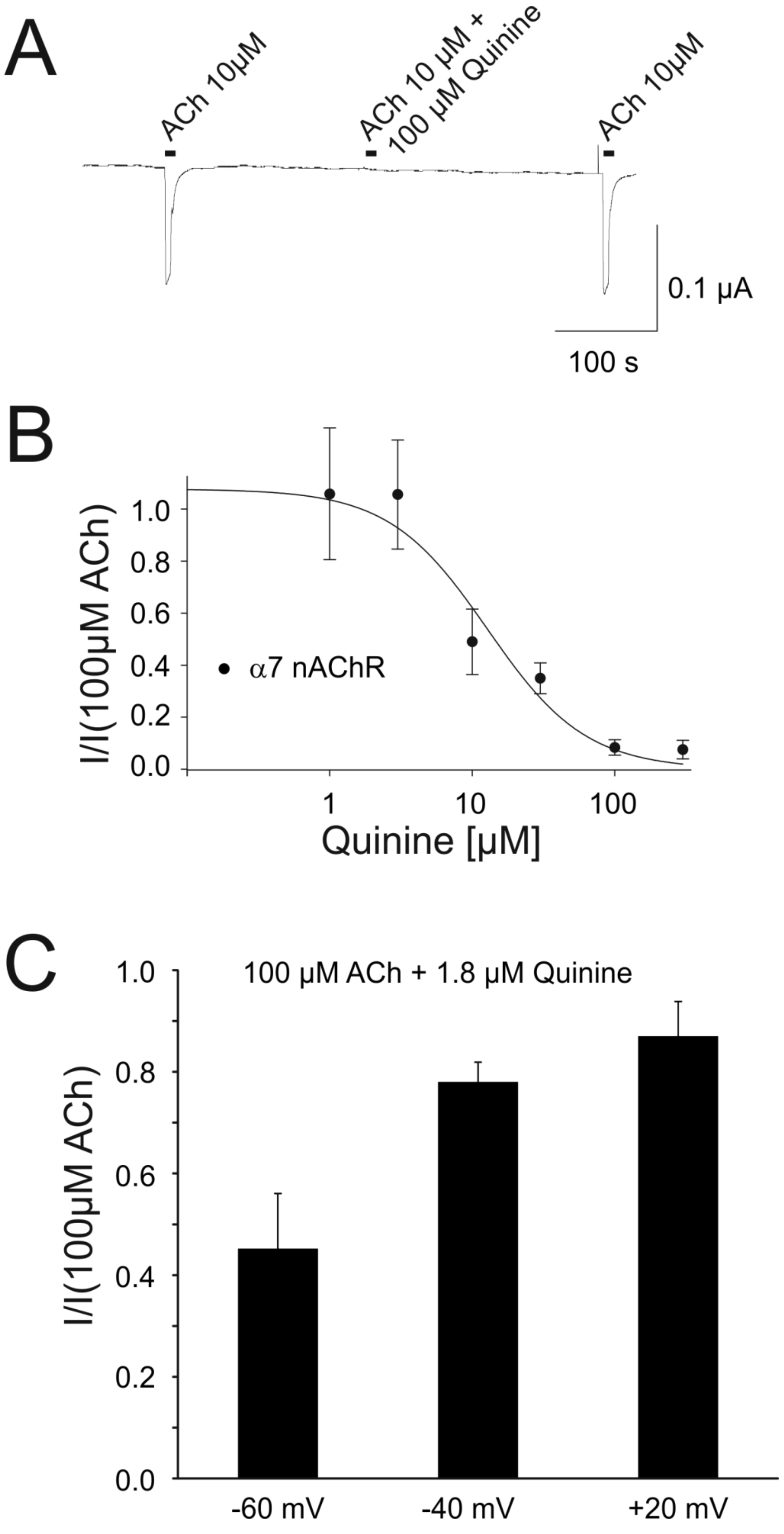
A
ACh 10μM
ACh 10 μM +
100 μM Quinine
ACh 10μM
0.1 μA
100 s
B
I/I(100μM ACh)
1.0
0.8
0.6
0.4
0.2
0.0
α7 nAChR
1
10
100
Quinine [μM]
C
100 μM ACh + 1.8 μM Quinine
I/I(100μM ACh)
1.0
0.8
0.6
0.4
0.2
0.0
-60 mV
-40 mV
+20 mV

Supplementary Fig. 1.

(A) Reversibility of the block of adult muscle nicotinic acetylcholine receptor by quinine in *Xenopus* oocytes. Membrane currents measured by two-electrode voltage-clamp. Currents were elicited by 10 µM ACh and blocked by 100 µM quinine in the second application. The quinine block was reversible as the third application of ACh evoked currents with a mean amplitude of 95.3±0.3% compared to the first ACh application (n=6).

(B) Block of the human neuronal α7 nicotinic acetylcholine receptor by quinine in *Xenopus* oocytes. Concentration–inhibition curves for quinine (circles) at ACh-mediated currents elicited by 100 µM ACh in the presence of different concentrations of the blocker. Quinine blocked the ACh-evoked currents in a dose-dependent manner with an IC_{50} of 12.8 ± 1.3 µM (n=3). Holding potential: -60 mV, error bars represent S.E.M.

(C) Voltage-dependency of the block of adult muscle nicotinic acetylcholine receptor by quinine in *Xenopus* oocytes. Currents were elicited by 100 µM ACh and blocked by 1.8 µM quinine in the second application. Measurements were performed at different membrane potentials. Quinine was significantly ($p<0.05$, n=3) more effective at a membrane potential of -60 mV compared to -40 mV and a positive membrane potential of 20 mV. Error bars represent S.E.M.
